# Distinguishing math learning and evaluation anxiety: a structural equation modeling study on pathways from teacher support to academic buoyancy

**DOI:** 10.3389/fpsyg.2026.1829556

**Published:** 2026-06-17

**Authors:** Yechao Sun, Yangyang Bai

**Affiliations:** College of Teacher Education, Ningbo University, Ningbo, China

**Keywords:** academic buoyancy, coping styles, math evaluation anxiety, math learning anxiety, teacher support behavior

## Abstract

**Introduction:**

While building academic resilience is crucial for adolescents facing intense educational demands, the specific mechanisms buffering subject-specific setbacks remain underexplored. This study examines the associations between math teacher support and high school students’ academic buoyancy within the highly demanding context of mathematics learning.

**Methods:**

Utilizing a cross-sectional survey with convenience sampling (N=411) from two cities, we first established measurement validity via confirmatory factor analysis (CFA), and subsequently employed structural equation modeling (SEM) to test competing models.

**Results:**

The results indicated that process-oriented math learning anxiety negatively predicted buoyancy, whereas outcome-oriented evaluation anxiety did not, identifying content anxiety as a core risk factor. Furthermore, teacher support positively predicted buoyancy directly and indirectly through three pathways: by promoting problem-focused coping, and via two distinct chain pathways associated with reduced learning anxiety through adaptive coping strategies. Importantly, the pathway linking teacher support to academic buoyancy via problem-focused coping was significantly stronger than the anxiety-reduction pathways.

**Discussion:**

These findings suggest that teacher support relates to enhanced resilience primarily through its association with adaptive coping strategies and reduced content anxiety. Consequently, educational interventions may benefit from addressing content-based cognitive difficulties and cultivating proactive problem-solving abilities over merely alleviating grade-related concerns.

## 1 Introduction

Globally, the mental health of adolescents and their strategies for coping with academic stress have become central issues in educational psychology. According to the PISA 2022 results released by the OECD (2023), with the intensification of global educational competition, ensuring students’ psychological well-being while maintaining high levels of academic performance has become a key indicator of the resilience of education systems. The high school stage, in particular, is a peak period for academic pressure and progression anxiety, during which students are more likely to encounter daily setbacks and challenges in their learning. To address this, researchers have increasingly focused on academic buoyancy, which refers to a student’s capacity to cope with typical, everyday academic setbacks and challenges (e.g., poor grades on routine tasks, high task difficulty, tight assignment deadlines) (Martin and Marsh, 2008).

Unlike traditional resilience paradigms that focus on major life adversity, academic buoyancy targets the common, everyday stressors faced by all students. Research indicates that everyday academic stress is a stronger predictor of individual psychological problems than major life events (Kanner et al., 1981). Enhancing academic buoyancy is not only crucial for students’ academic success (Martin et al., 2010; Putwain and Daly, 2013; Collie et al., 2015) but is also associated with adaptive psychological outcomes such as life satisfaction and self-esteem (DiCorcia and Tronick, 2011; Martin et al., 2013). Consequently, high school students’ ability to successfully navigate these daily academic setbacks significantly shapes their academic and even life trajectories (Putwain et al., 2024).

While the “academic” component of academic buoyancy has often been conceptualized holistically as a general construct, high school students typically exhibit subject-specific academic strengths and weaknesses (Wang and Lu, 2022). Research confirms significant variations in students’ academic self-concepts across different disciplines, which are formed through internal comparisons and linked to differentiated self-efficacy (Marsh et al., 2001; Möller et al., 2006; Stocker et al., 2021). Because academic self-concept is directly tied to students’ expectations of challenges, persistence, and choice of coping strategies when facing difficulties (Marsh et al., 1988; Marsh, 1990), individuals may naturally exhibit varying levels of academic buoyancy depending on the specific subject context. Among high school subjects, mathematics is uniquely characterized by high abstraction, rigorous logic, and a cumulative knowledge structure (Ministry of Education of the People’s Republic of China, 2018). These specific traits render mathematics a high-incidence area for daily academic setbacks. Given that knowledge gaps in mathematics often trigger immediate and persistent content-related difficulties (Fulano et al., 2020), the coping strategies students adopt in response to these setbacks have far-reaching consequences, impacting not only their immediate academic performance but also their long-term career trajectories and lifelong learning (Huang et al., 2019). Therefore, investigating academic buoyancy specifically within the context of mathematics learning is necessary to accurately capture how students recover from these distinct, everyday cognitive challenges.

However, to accurately capture this recovery process, a critical theoretical gap must be addressed. Previous research often treats ‘math anxiety’ as a unitary construct, which obscures the distinct emotional mechanisms at play. In the highly competitive high school environment, it remains unclear whether students’ daily buoyancy is primarily undermined by process-oriented math learning anxiety (the cognitive struggle of understanding mathematical content) or outcome-oriented math evaluation anxiety (the pressure of testing). Explicitly distinguishing between these two anxieties is a core innovation of the present study, aiming to pinpoint the primary emotional risk factor.

Given the profound impact of these mathematical setbacks, building academic buoyancy is rarely a solitary endeavor for high school students; rather, it often requires the mobilization of external protective resources. The school environment is a crucial exogenous factor influencing academic buoyancy (Martin and Marsh, 2008), with teacher support serving as a core component. Although much of the existing research on teacher support and resilience has been conducted across general academic domains, these protective mechanisms are particularly vital in a demanding subject like mathematics. Perceived math teacher support encompasses emotional, autonomy, and cognitive support (Skinner and Belmont, 1993; Lam et al., 2009). By providing a positive emotional atmosphere, clear instructional guidance, and immediate feedback, such multidimensional support creates a safe psychological buffer that enables students to maintain a positive psychological state and mobilize coping resources when encountering mathematical setbacks (Werner, 2000; Furrer et al., 2014; Hu, 2021; Liu, 2023). Within the “protective factor model” framework, an individual’s adaptive development is shaped by the complex interplay of protective factors and risk factors (Rutter, 1987; Masten et al., 1990). The true value of protective factors like teacher support lies not merely in their independent existence, but rather in their capacity to buffer or transform internal risk factors, such as anxiety (Zhao and Yu, 2018). Therefore, it is essential to investigate the specific mechanisms through which math teacher support fosters academic buoyancy when risk and protective factors co-occur.

### 1.1 Theoretical framework and conceptual model

The present study is grounded in the protective factor model, which posits that an individual’s adaptive development is shaped by the dynamic interplay between external protective resources and internal risk factors (Garmezy et al., 1984). Within this overarching framework, math teacher support is conceptualized as the external protective resource, while academic buoyancy represents the adaptive outcome in response to everyday setbacks. A core tenet of this model is that the value of protective factors lies in their capacity to buffer or transform the negative impacts of risk factors, rather than simply eliminating them.

To elucidate the specific psychological pathways through which this protective process occurs, the current study integrates Self-Determination Theory (SDT) (Deci and Ryan, 2000; Reeve, 2012), coping theory, and Control-Value Theory (CVT) (Pekrun et al., 2007; Pekrun, 2024) into a cohesive conceptual model. Specifically, SDT provides a motivational perspective, explaining how teacher support functions as a protective resource by satisfying students’ basic psychological needs. Coping theory delineates the behavioral regulation mechanisms, detailing how perceived support shapes students’ choice of coping strategies when facing mathematics-related challenges. Finally, CVT accounts for the emotional mechanisms, articulating how these behavioral responses influence subject-specific achievement emotions, specifically the internal risk of math anxiety, which is closely linked to the level of academic buoyancy. By nesting these theories within the protective factor model, we construct a theoretically ordered process from external support to behavioral and emotional regulation.

Within this framework, Self-Determination Theory (SDT) provides a fundamental explanation for how math teacher support functions as a protective resource. Math teacher support, encompassing emotional, autonomy, and cognitive dimensions, is crucial for fostering students’ academic buoyancy (Skinner and Belmont, 1993; Lam et al., 2009). Specifically, emotional support from teachers conveys acceptance and care, thereby satisfying students’ need for relatedness and enhancing their sense of belonging within the mathematics classroom (Romano et al., 2021; Shen et al., 2024). Cognitive and autonomy support, characterized by clear instructional guidance and the promotion of student agency, fulfill the needs for competence and autonomy, respectively (Deci and Ryan, 2000; Reeve, 2012). The satisfaction of these basic psychological needs strengthens students’ learning self-efficacy and perceived control, which in turn facilitates their confidence in overcoming subject-specific setbacks and promotes sustained academic re-engagement (Zhao and Yu, 2018; Lei et al., 2022; Kang et al., 2024). Thus, teacher support serves as a motivational catalyst that empowers students to persist when facing the high cognitive demands and daily challenges inherent in mathematics learning.

Building upon the motivational foundation established by SDT, coping theory and Control-Value Theory (CVT) delineate the subsequent behavioral and emotional pathways within the protective factor model. According to the contextual model of coping (Lazarus and Folkman, 1984), an individual’s appraisal of whether a stressful situation is changeable significantly dictates their preferred coping response. High levels of perceived teacher support can bolster students’ beliefs in their capacity to alter adverse academic situations, encouraging the adoption of constructive problem-focused coping over ineffective emotion-focused coping (He, 2012; Song and Li, 2020; Zhang, 2023). Furthermore, CVT posits that these appraisals of control and value in academic contexts fundamentally shape students’ achievement emotions (Pekrun et al., 2007; Pekrun, 2024). In the demanding context of mathematics, the proactive behavioral shift facilitated by teacher support directly addresses cognitive barriers, mitigating the negative emotional experiences associated with math anxiety. Consequently, the integration of coping theory and CVT bridges the gap between external support and internal resilience, demonstrating that teacher support safeguards academic buoyancy not merely by providing comfort, but by fundamentally reshaping students’ behavioral strategies and emotional risk profiles in the face of daily setbacks.

### 1.2 Unpacking the mechanisms: coping styles and anxiety subtypes

As delineated in the overarching theoretical framework, behavioral regulation serves as the critical bridge between external support and individual academic outcomes. In the context of mathematics learning, problem-focused coping involves proactive efforts to address the source of academic stress, such as actively seeking help for difficult problems, adjusting learning strategies, and formulating effective study plans (Lazarus and Folkman, 1984; Zhang, 2023). This strategy aims to overcome the cognitive barriers inherent in the process of acquiring mathematical knowledge. Research consistently demonstrates that this proactive approach is positively associated with academic buoyancy, as it directly removes cognitive barriers and builds the necessary competence to navigate future setbacks (Martin and Marsh, 2008; Putwain et al., 2024). Conversely, emotion-focused coping aims primarily to regulate negative emotional states without altering the actual academic stressor, often manifesting as avoidance, denial, or emotional venting (He, 2012; Song and Li, 2020). While such strategies may offer temporary psychological relief, they fail to resolve the underlying knowledge gaps. In a cumulative subject like mathematics, relying on emotion-focused coping often exacerbates subsequent learning difficulties, thereby impeding the development of academic buoyancy and predicting maladaptive outcomes. Thus, these two coping styles represent fundamentally divergent behavioral pathways in the face of daily mathematical challenges.

Beyond behavioral regulation, students’ subjective emotional states, specifically math anxiety, serve as a critical internal risk factor that compromises academic buoyancy. In highly competitive high school environments, students frequently confront a dual challenge: the high cognitive load inherent in the daily construction of mathematical knowledge and the intense pressure of high-stakes evaluations. Consequently, students often experience both math learning anxiety and math evaluation anxiety simultaneously. Distinguishing between these two constructs is essential because they represent fundamentally different psychological risks; a generalized measurement of “math anxiety” obscures whether a student’s vulnerability stems from the daily process of understanding content or the outcome of assessments. Based on the two-factor model proposed by Hopko et al. (2003), math learning anxiety pertains to the tension experienced during the daily acquisition and processing of mathematical knowledge, reflecting a fear of the mathematical content itself in concrete learning contexts (e.g., listening to lectures, solving problems) (Ashcraft, 2002; Commodari and La Rosa, 2021). Conversely, math evaluation anxiety is the distress experienced in testing situations, primarily driven by the worry that one’s grades will not meet personal or others’ expectations (Lanfaloni et al., 2023). Crucially, to accurately identify the core mechanism undermining psychological recovery from daily setbacks, these two distinct anxieties must be included in the conceptual model simultaneously. Treating them as a single construct or examining them in isolation fails to account for their shared variance, which could yield spurious statistical conclusions regarding their true destructive effects. By integrating them into a unified, competitive analytical framework, this study can better isolate their independent associations with academic buoyancy when they co-occur.

The distinct cognitive mechanisms of math learning anxiety and math evaluation anxiety suggest they may differentially disrupt the process of building academic buoyancy. According to the Encoding Deficit Hypothesis (EDH) (Eysenck and Calvo, 1992), math learning anxiety primarily interferes with the initial encoding of information into long-term memory during the daily learning process (Prevatt et al., 2010; Zhu et al., 2024). When students experience tension due to difficulties in understanding content, this anxiety consumes limited working memory resources, potentially triggering a reciprocal cycle of “anxiety—avoidance—poor performance” (Carey et al., 2016). This high-frequency psychological depletion aligns closely with the temporal dimension of everyday academic setbacks, which are the central focus of academic buoyancy (Zhao and Yu, 2018). In contrast, math evaluation anxiety, while also inducing “cognitive interference” according to Attentional Control Theory (ACT) (Eysenck et al., 2007; Derakshan and Eysenck, 2009), is often characterized as situational or episodic. Its impact typically peaks during high-stakes assessments, potentially acting as a disturbance to the externalization of existing knowledge rather than the foundational daily construction of learning. Consequently, while theoretical deductions imply that the chronic nature of learning anxiety might be more detrimental to daily academic buoyancy than episodic evaluation anxiety, this hypothesis awaits direct empirical verification when both risks co-occur. This underscores the necessity of the current inquiry: empirically investigating which specific anxiety subtype constitutes the core mechanism linking behavioral coping to academic buoyancy in the face of daily mathematical setbacks.

### 1.3 The current study

Taken together, although existing research has established a general link between environmental support and students’ academic resilience, critical gaps remain. First, empirical studies on academic buoyancy have predominantly focused on general school contexts or language learning (Hu, 2021; Liu, 2023). Despite the consensus that resilience is highly subject-specific (Marsh et al., 2001; Yun et al., 2018; Hu, 2021), the unique cognitive and emotional challenges inherent in mathematics remain underexplored. Second, regarding the emotional mechanisms, prior literature often treats math anxiety as a unitary construct. This generalized approach fails to disentangle the differential impacts of process-oriented learning anxiety versus outcome-oriented evaluation anxiety on daily adaptation (Pizzie and Kraemer, 2019; Lanfaloni et al., 2023). Finally, while the protective factor model provides a foundational framework (Garmezy et al., 1984), the field lacks an integrated investigation that elucidates the sequential pathways from external support to behavioral coping, and subsequently to specific emotional regulation. Consequently, the current study constructs a competitive chain mediation model to address these gaps, aiming to clarify how math teacher support fosters academic buoyancy in high school students.

To address these gaps, the present study aims to investigate the psychological mechanisms through which math teacher support influences high school students’ academic buoyancy. Specifically, we employ structural equation modeling (SEM) to test a theoretically ordered chain mediation model grounded in the protective factor framework. Furthermore, rather than arbitrarily selecting one type of anxiety or examining them in isolation, we construct competing path models to test math learning anxiety and math evaluation anxiety simultaneously. This competitive modeling strategy is deliberately chosen not to unnecessarily complicate the theoretical framework, but to rigorously control for shared variance and identify the core emotional risk factor that primarily compromises daily academic resilience.

**Figure 1:**
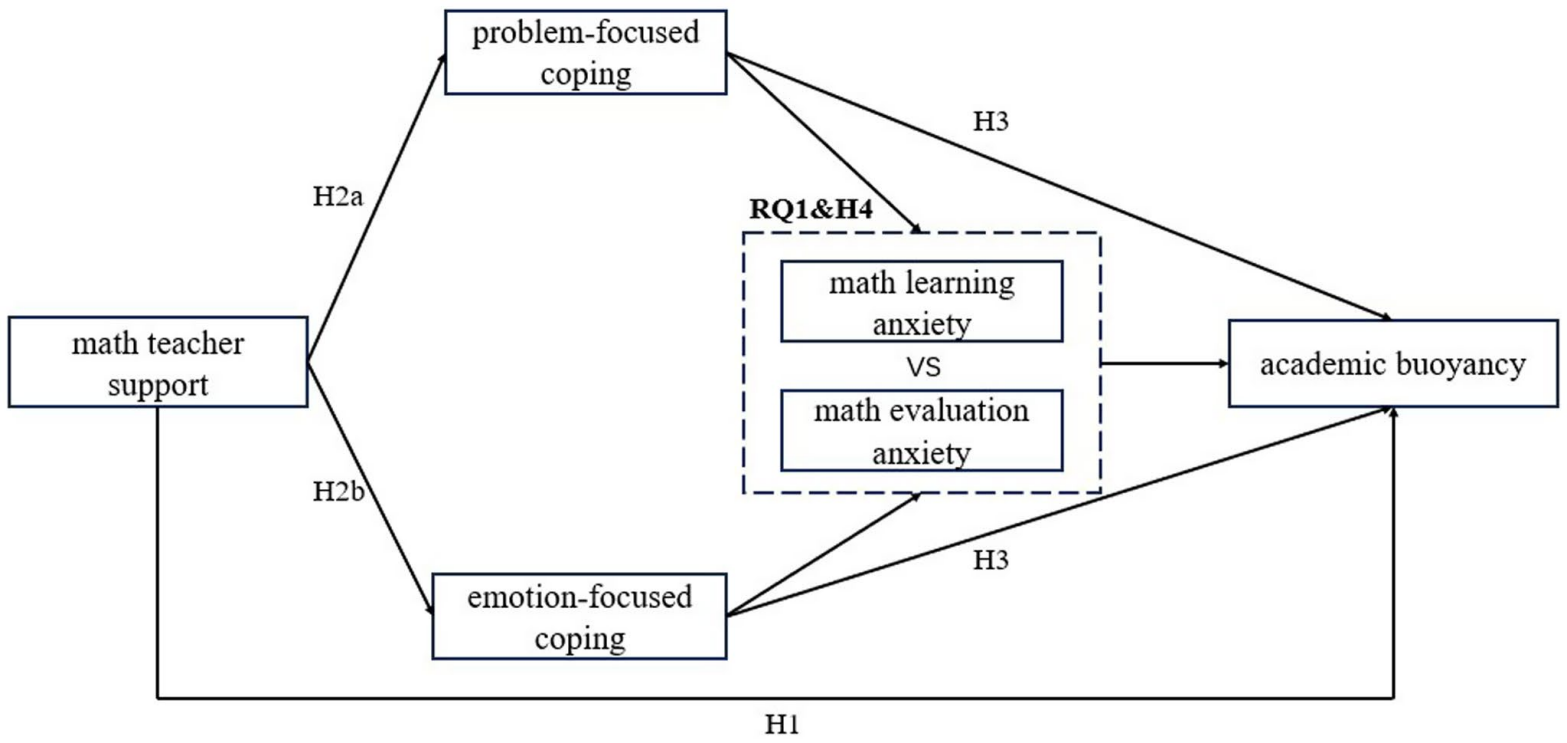
The hypothesized conceptual model of the current study.

Therefore, the primary objective of this research is to examine the relationship between math teacher support and students’ academic buoyancy, and to elucidate the underlying psychological mechanisms. Based on the theoretical integration and empirical evidence discussed above, this study proposes the following hypotheses and research question (Figure 1):

*Hypothesis 1 (H1)*: Math teacher support positively predicts academic buoyancy.

*Hypothesis 2 (H2)*: Math teacher support positively predicts problem-focused coping (H2a) and negatively predicts emotion-focused coping (H2b).

*Hypothesis 3 (H3)*: Coping styles mediate the relationship between math teacher support and academic buoyancy.

*Research Question 1 (RQ1)*: In the context of daily mathematics learning, which anxiety subtype (process-oriented learning anxiety or outcome-oriented evaluation anxiety) serves as the core risk mechanism connecting coping styles to academic buoyancy?

*Hypothesis 4 (H4)*: Coping styles and the core math anxiety (determined by RQ1) sequentially mediate the relationship between math teacher support and academic buoyancy.

## 2 Method

### 2.1 Participants

The participants in this study were 450 high school students from City W and City Z in Province Z. To ensure practical feasibility within the natural school environment, this study employed a class-based convenience sampling strategy. With the assistance of school administrators and mathematics teachers, intact classes in Grade 10 and Grade 11 were invited to participate during their regular self-study sessions. Prior to the commencement of the study, the research protocol was approved by the ethics committee, and written informed consent was obtained from all participating students and their guardians. A total of 450 students were initially approached. After excluding invalid responses (e.g., incomplete questionnaires, obvious patterned answering), 411 valid questionnaires were recovered, yielding an effective response rate of 91.3%. Among the participants, 57.7% were in Grade 10 and 42.3% were in Grade 11. In terms of gender distribution, there were 196 boys and 215 girls. The participants’ ages ranged from 15 to 17 years, with a mean age of 15.92 years (SD = 0.71).

### 2.2 Measures

#### 2.2.1 Math teacher support

The Math Teacher Support Scale (MTSS) for middle school students, developed by Chai and Gong (2013), was adopted. To minimize survey fatigue and accommodate the cognitive load of high school students, an abbreviated version of the scale consisting of 5 core items was administered in this study. Furthermore, to optimize the measurement model for structural equation modeling (SEM) and ensure the most robust representation of the construct, an item purification process was conducted based on the initial Confirmatory Factor Analysis (CFA). Ultimately, 3 highly representative items with strong standardized factor loadings were retained to form a unidimensional measure of math teacher support. A 7-point Likert scale was used, with higher scores indicating higher levels of perceived math teacher support. In this study, the Cronbach’s *α* coefficient for the questionnaire was 0.844.

#### 2.2.2 Coping styles

The Coping Style Scale (CSS) for Middle School Students, developed by Chen et al. (2000), was adopted. As this scale was developed based on the specific developmental stage and cultural background of Chinese middle school students (Huang et al., 2000), it was deemed appropriate for this study. The questionnaire comprises 36 items divided into two main dimensions: problem-focused coping and emotion-focused coping. To align with the specific context of this study, a prompt regarding the mathematics context was added before the relevant items, and this was re-emphasized by the examiner during administration. A 7-point Likert scale was used, with higher scores indicating a greater frequency of using that coping style. In this study, the Cronbach’s *α* coefficients for the problem-focused and emotion-focused coping scales were 0.898 and 0.784, respectively.

#### 2.2.3 Math learning anxiety

The Abbreviated Math Anxiety Scale (AMAS) developed by Hopko et al. (2003) was adopted. Specifically, the “Math Learning Anxiety” dimension was selected as its definition aligns with the operational definition of math anxiety in this study. This subscale consists of 5 items (e.g., “Listening to a lecture in math class”). A 7-point Likert scale was used, with higher scores indicating higher levels of math anxiety. In this study, the Cronbach’s α coefficient for this questionnaire was 0.786.

#### 2.2.4 Academic buoyancy

The Academic Buoyancy Scale (ABS) developed by Martin and Marsh (2008) was adopted. The questionnaire consists of 4 items, modified to include a mathematics context (e.g., “I don’t let study stress get on top of me” was changed to “I don’t let math study stress get on top of me”). A 7-point Likert scale was used, with higher scores indicating higher levels of academic buoyancy. In this study, the Cronbach’s α coefficient for this questionnaire was 0.890.

#### 2.2.5 Math evaluation anxiety

The Abbreviated Math Anxiety Scale (AMAS) developed by Hopko et al. (2003) was adopted. The “Math Evaluation Anxiety” dimension consists of 4 items (e.g., “Thinking about an upcoming math test 1 day before”). A 7-point Likert scale was used, with higher scores indicating higher levels of math evaluation anxiety. In this study, the Cronbach’s α coefficient for this subscale was 0.899.

### 2.3 Data analysis

Data processing and analysis were conducted using SPSS 26.0 and Amos 24.0. The analytic procedure consisted of four main stages. First, preliminary analyses were conducted to handle missing data and evaluate common method bias (CMB). Second, prior to conducting Structural Equation Modeling (SEM), statistical assumptions were verified. This included assessing univariate normality via skewness and kurtosis, and testing for multicollinearity utilizing tolerance and variance inflation factors (VIF). Third, Confirmatory Factor Analysis (CFA) was employed to evaluate the reliability and validity of the measurement model. Fourth, SEM with Maximum Likelihood (ML) estimation was utilized to test the hypothesized and competing structural models. The Bootstrap method (with 5,000 resamples) was used to test the significance of direct and indirect effects.

Model fit was systematically evaluated using multiple indices, including *χ*^2^/*df*, Comparative Fit Index (CFI), Goodness of Fit Index (GFI), Adjusted Goodness of Fit Index (AGFI), Root Mean Square Error of Approximation (RMSEA), and Standardized Root Mean Square Residual (SRMR). In the presence of competing structural models, the final model selection was based on a combination of statistical fit criteria and the principle of theoretical parsimony.

## 3 Results

### 3.1 Preliminary analysis and common method bias test

Prior to the main structural equation modeling, descriptive statistics and statistical assumptions were evaluated. First, the univariate normality of the primary constructs was assessed. According to Kline (2023), variables with an absolute skewness value less than 3.0 and an absolute kurtosis value less than 8.0 sufficiently approximate a normal distribution. In this study, the absolute values of skewness ranged from 0.266 to 0.999, and kurtosis ranged from 0.122 to 0.840. Furthermore, to robustly address any potential deviations from strict multivariate normality, maximum likelihood (ML) estimation was employed in conjunction with a bootstrapping procedure (5,000 resamples). Bootstrapping provides robust parameter estimates and standard errors that do not rely on the assumption of multivariate normality (Byrne, 2016).

Then, potential common method bias (CMB) was evaluated. Harman’s single-factor test was employed to check for common method bias. An exploratory factor analysis (EFA) was conducted on all items. The results showed that there were 5 factors with eigenvalues greater than 1. The first factor explained 33.64% of the variance, which is below the critical threshold of 40% (Ylitalo, 2009). While Harman’s single-factor test provides an initial assessment, an unmeasured latent method construct (ULMC) approach was further employed in the Confirmatory Factor Analysis (CFA) to rigorously control for common method bias (CMB). A common latent factor was introduced to the baseline 6-factor CFA model, with equally constrained paths directed to all 19 observed items. The results demonstrated that the inclusion of the ULMC did not yield any substantive improvement in model fit (△CFI = 0.001, △RMSEA = 0.000). The fit indices of the ULMC model remained virtually identical to the baseline model (*χ*^2^/*df* = 1.817, CFI = 0.976, RMSEA = 0.045). Furthermore, the standardized factor loadings of the ULMC on all items were close to zero, while the loadings on their respective trait factors remained robust and stable. These findings suggest that common method bias is unlikely to be a major concern the validity of the current study.

### 3.2 Reliability and validity test of variables

Regarding the cultural adaptation of the instruments, all scales utilized in this study have been previously translated, adapted, and validated for use with Chinese adolescent populations in prior literature.

To optimize the measurement model and maintain an acceptable indicator-to-sample-size ratio for SEM, an item purification and reduction process was systematically conducted. Given that including all original items (e.g., the 36 items of the Coping Style Scale) would introduce excessive model complexity and potential cross-loadings, we retained only the most representative items for each latent construct. Specifically, items with low standardized factor loadings (< 0.50) or significant cross-loadings were iteratively removed. Ultimately, 3 to 4 robust indicators were retained for each latent variable (except for Math Teacher Support, which utilized the aforementioned domain-level parceling). After this rigorous item reduction, the final CFA model consisted of 19 indicators. A poor measurement model can lead to erroneous judgments regarding the existence, magnitude, and direction of associations between constructs. Therefore, a Confirmatory Factor Analysis (CFA) was conducted on the dimensions of the model prior to the formal Structural Equation Modeling (SEM) analysis. The overall fit indices of the CFA were: *χ*^2^ = 247.152, *df* = 137, *χ*^2^/*df* = 1.804, GFI = 0.944, AGFI = 0.922, CFI = 0.976, RMSEA = 0.044, and SRMR = 0.0439. The results of the reliability and validity analysis for each dimension are presented in Table 1, and the discriminant validity is shown in Table 2.

**Table 1:** Reliability and validity test of variables.

Item	Construct	Parameter estimates				Factor Loading	Item reliability	Composite reliability	Convergent validity
		Unstd.	S.E.	*t*-value	*p*	Std.	SMC	CR	AVE
TSM	TSM1	1.000				0.855	0.731	0.851	0.656
	TSM2	1.036	0.063	16.487	***	0.825	0.681		
	TSM3	1.047	0.068	15.445	***	0.745	0.555		
EC	EC1	1.000				0.662	0.438	0.787	0.554
	EC2	1.304	0.116	11.264	***	0.825	0.681		
	EC3	1.180	0.103	11.452	***	0.736	0.542		
PC	PC1	1.000				0.861	0.741	0.901	0.752
	PC2	1.191	0.055	21.637	***	0.874	0.764		
	PC3	1.022	0.048	21.475	***	0.867	0.752		
MLA	MLA1	1.000				0.681	0.464	0.791	0.559
	MLA2	1.036	0.089	11.661	***	0.808	0.653		
	MLA3	1.016	0.087	11.739	***	0.748	0.560		
AB	AB1	1.000				0.915	0.837	0.897	0.747
	AB2	1.080	0.041	26.625	***	0.960	0.922		
	AB3	0.650	0.038	17.300	***	0.695	0.483		
MEA	MEA1	1.000				0.855	0.731	0.900	0.692
	MEA2	0.924	0.049	18.946	***	0.793	0.629		
	MEA3	1.062	0.049	21.869	***	0.879	0.773		
	MEA4	0.899	0.047	19.090	***	0.797	0.635		

TSM, Math Teacher Support; MLA, Math Learning Anxiety; PC, Problem-Focused Coping; EC, Emotion-Focused Coping; AB, Academic Buoyancy; MEA, Math Evaluation Anxiety.

**Table 2:** Analysis of discriminant validity.

Construct	No. of items	Discriminant validity					
		TSM	MLA	PC	EC	AB	MEA
TSM	3	0.810					
MLA	3	−0.194	0.748				
PC	3	0.368	−0.296	0.867			
EC	3	−0.162	0.327	−0.461	0.744		
AB	3	0.423	−0.623	0.483	−0.292	0.864	
MEA	4	−0.138	0.725	−0.127	0.128	−0.49	0.832

TSM, Math Teacher Support; MLA, Math Learning Anxiety; PC, Problem-Focused Coping; EC, Emotion-Focused Coping; AB, Academic Buoyancy; MEA, Math Evaluation Anxiety.

As indicated in the table, the square roots of the Average Variance Extracted (AVE) for all constructs (the values on the diagonal) are greater than the correlation coefficients in their respective rows and columns. This indicates that all constructs in the model have good discriminant validity. It is worth noting that the correlation coefficient between the two constructs of math learning anxiety and math evaluation anxiety is relatively high (0.725). To further rule out potential collinearity issues, this study additionally employed the Heterotrait-Monotrait Ratio (HTMT) method for verification. The results showed that the HTMT value between math learning anxiety and math evaluation anxiety was 0.729. This value is below the widely accepted threshold of 0.85 (Henseler et al., 2015), indicating that although these two variables are highly correlated, they remain conceptually distinct constructs with good discriminant validity. To further verify the absence of severe multicollinearity at the structural level, Variance Inflation Factor (VIF) and tolerance values were computed. The VIF values for all predictors ranged from 1.121 to 1.753, which are strictly below the conservative threshold of 5.0. Correspondingly, the tolerance values ranged from 0.571 to 0.892, exceeding the 0.10 minimum. These dual diagnostics (HTMT and VIF) provide robust evidence that multicollinearity does not compromise the independence or the estimates of the subsequent structural equation models.

### 3.3 Model analysis with math evaluation anxiety as the mediating variable

To robustly test the distinct roles of math evaluation anxiety (MEA) and math learning anxiety (MLA) and avoid potential multicollinearity issues (*r* = 0.725), we adopted a stepwise competitive modeling strategy. First, we tested a comprehensive structural model with MEA as the primary mediator, controlling for MLA, to evaluate its theoretical viability. If this model failed to demonstrate adequate fit or significant pathways, subsequent isolation tests would be conducted to directly pit the two anxieties against each other.

**Figure 2:**
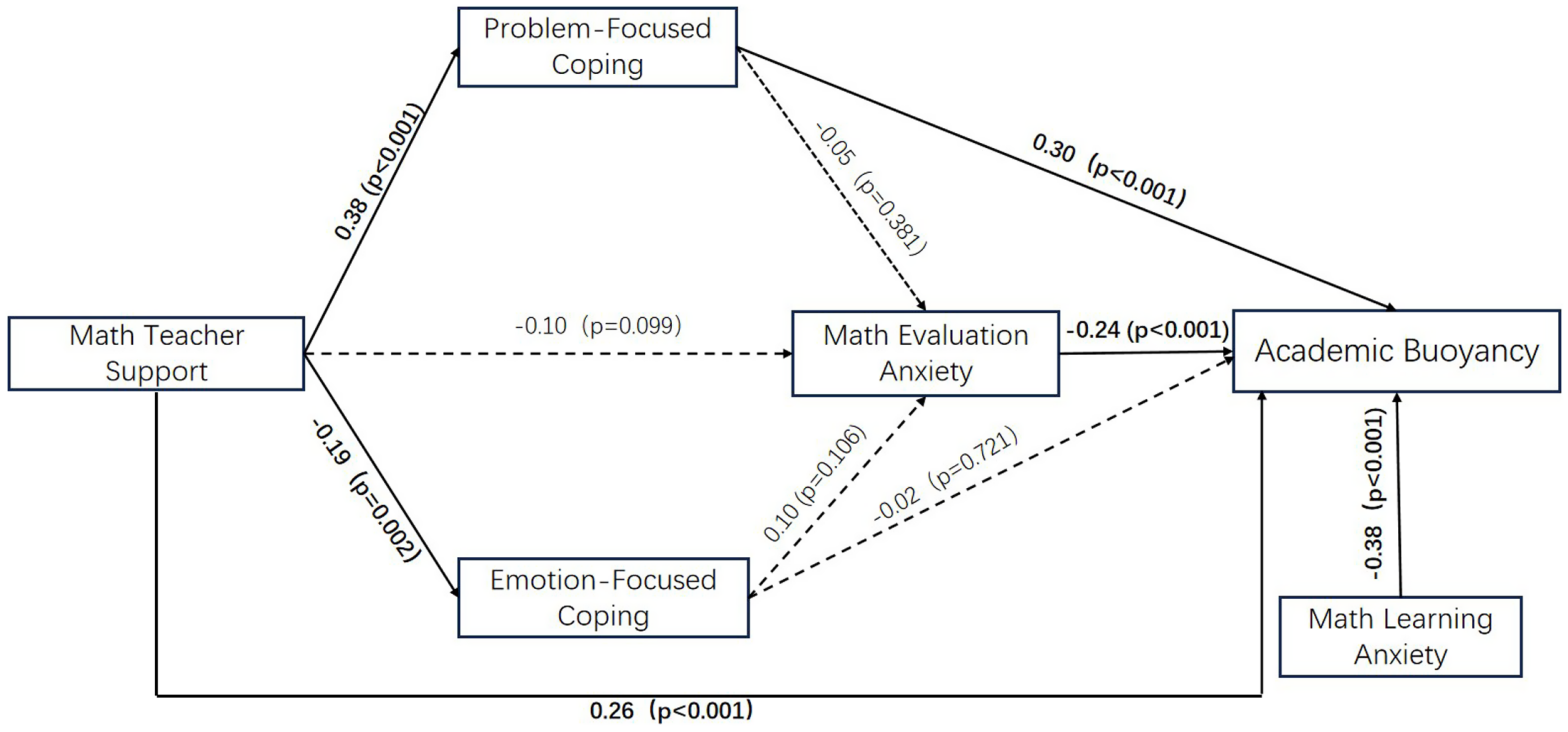
Results of the model with math evaluation anxiety as the mediating variable and math learning anxiety as a control variable.

The results showed that the fit indices for the model with math evaluation anxiety as the mediator were *χ*^2^ = 534.704, *df* = 142, *χ*^2^/*df* = 3.766, GFI = 0.887, AGFI = 0.848, CFI = 0.914, RMSEA = 0.082, and SRMR = 0.1609. These fit indices, particularly the SRMR far exceeding the 0.08 threshold and the RMSEA above 0.08, provide clear evidence of model misspecification (results are shown in Figure 2). This indicates that the hypothesized structural relations involving MEA as a mediator do not adequately represent the observed covariance structure of the data. Furthermore, several key predictive paths from math teacher support and coping styles to MEA were non-significant (*p* > 0.05). Given this, we rejected this model and preliminarily conjectured that math evaluation anxiety is not the core mediating mechanism explaining how teacher support influences academic buoyancy.

**Figure 3:**
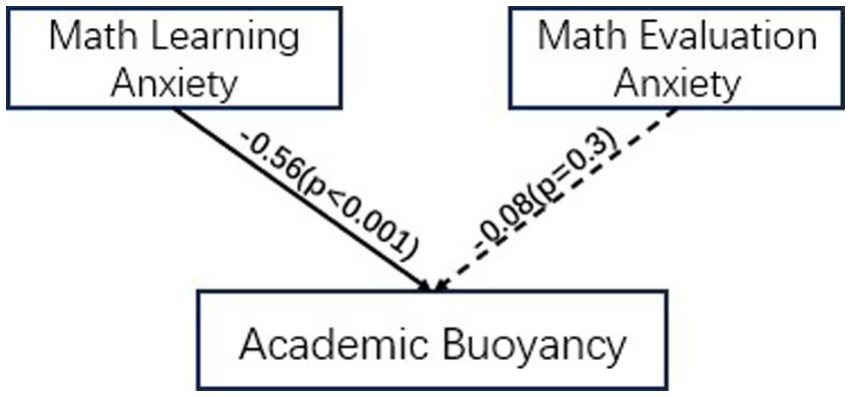
Simplified model.

Although the initial comprehensive model yielded poor overall fit indices, the internal path from math evaluation anxiety to academic buoyancy appeared significant (*β* = −0.24, *p* < 0.001). To verify whether this specific path was a robust finding or merely a spurious artifact caused by potential suppression effects within the complex structural network, an isolation model (simplified model) was subsequently constructed (see Figure 3). This simplified model, which demonstrated excellent fit statistics (*χ*^2^ = 85.657, *df* = 32, *χ*^2^/*df* = 2.677, GFI = 0.962, AGFI = 0.935, CFI = 0.979, RMSEA = 0.064), provided a direct, head-to-head comparison of the independent predictive utility of both math evaluation anxiety and math learning anxiety on academic buoyancy, free from upstream confounding variables.

Synthesizing the results from these two models, we have sufficient and multifaceted evidence to demonstrate that math evaluation anxiety and math learning anxiety are conceptually distinct, and that math evaluation anxiety is not a core variable within the theoretical framework of this study. Consequently, its exclusion from the construction of the final theoretical model is both reasonable and justified.

### 3.4 Model analysis with math learning anxiety as the mediating variable

**Figure 4:**
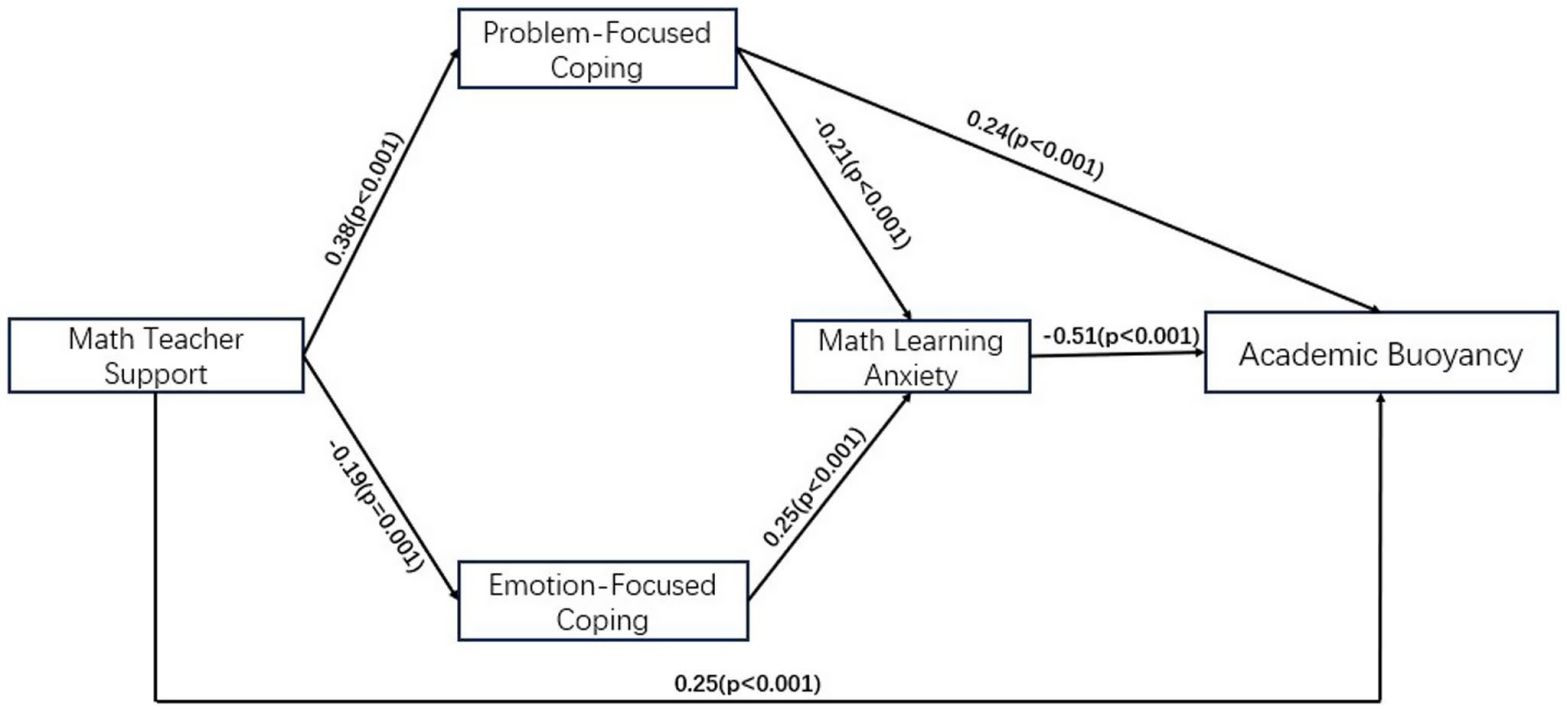
Schematic of the revised model.

The results of the isolation test indicated that that MLA accounted for the majority of the predictive variance of MEA on academic buoyancy. In structural equation modeling, the principle of model parsimony dictates that non-significant and redundant pathways should be trimmed to achieve the most robust and theoretically meaningful model. Consequently, MEA was excluded from the final theoretical framework. Based on this empirically driven justification, the model was respecified with MLA as the core mediating variable, as illustrated in Figure 4. The fit indices for this model were *χ*^2^ = 203.258, *df* = 83, *χ*^2^/*df* = 2.449, GFI = 0.938, AGFI = 0.910, CFI = 0.964, RMSEA = 0.059, SRMR = 0.085. These values indicate a high degree of construct validity, demonstrating the model’s suitability for subsequent path analysis and mediation effect testing.

The path analysis results indicated that the coefficients for all paths within the model reached statistical significance (see Table 3). Math learning anxiety demonstrated the strongest negative predictive effect on academic buoyancy (*β* = −0.51, *p* < 0.001). Meanwhile, the influence of math teacher support exhibited a dual-pathway characteristic involving both direct and indirect effects. It not only directly and positively predicted academic buoyancy (*β* = 0.25, *p* < 0.001) but also exerted its influence by significantly promoting problem-focused coping (*β* = 0.38, *p* < 0.001) and inhibiting emotion-focused coping (*β* = −0.19, *p* = 0.001).

**Table 3:** Path coefficients of the revised structural equation model.

DV	IV	Unstd. (B)	S.E.	C.R. (*t*)	*p*	Std. (*β*)	95% CI [Lower, Upper]
PC	TSM	0.352	0.051	6.835	***	0.380	[0.249, 0.473]
EC	TSM	−0.233	0.072	−3.234	0.001	−0.192	[−0.386, −0.094]
MLA	PC	−0.290	0.081	−3.593	***	−0.208	[−0.498, −0.088]
MLA	EC	0.269	0.066	4.096	***	0.253	[0.097, 0.470]
AB	MLA	−0.639	0.069	−9.210	***	−0.507	[−0.782, −0.503]
AB	PC	0.427	0.084	5.108	***	0.243	[0.242, 0.620]
AB	TSM	0.406	0.077	5.247	***	0.250	[0.237, 0.577]

TSM, Math Teacher Support; MLA, Math Learning Anxiety; PC, Problem-Focused Coping; EC, Emotion-Focused Coping; AB, Academic Buoyancy. Unstandardized estimates, standard errors (S.E.), critical ratios (C.R.), and p-values are based on Maximum Likelihood estimation. The 95% Confidence Intervals (CI) are based on 5,000 bootstrap resamples.

Furthermore, the squared multiple correlations (*R*^2^) indicated that the revised structural model possessed strong explanatory power. Specifically, the model accounted for 14.4% of the variance in problem-focused coping, 3.7% in emotion-focused coping, and 11.5% in math learning anxiety. The integrated pathways explained 51.3% of the total variance in high school students’ academic buoyancy, demonstrating the substantial predictive validity of the theoretical framework.

### 3.5 Mediation effect test

This study employed the Bootstrap method (with 5,000 resamples) in Amos 24.0 to test for chain mediation effects. The results are presented in Table 4. The direct path from math teacher support to academic buoyancy was significant (Bias-corrected 95% CI [0.237, 0.577]), accounting for 61.4% of the total effect. The independent mediating effect of problem-focused coping was significant (Bias-corrected 95% CI [0.078, 0.254]), accounting for 22.7% of the total effect. The chain mediating effect of problem-focused coping and math anxiety between math teacher support and academic buoyancy was significant (Bias-corrected 95% CI [0.021, 0.127]), accounting for 9.8% of the total effect. The chain mediating effect of emotion-focused coping and math anxiety between math teacher support and academic buoyancy was also significant (Bias-corrected 95% CI [0.011, 0.091]), accounting for 6.1% of the total effect.

**Table 4:** Mediation effects and effect sizes.

Path	Bias-corrected 95%CI		Percentile 95%CI		Effect analysis	
	Lower	Upper	Lower	Upper	Effect	Proportion
TSM to AB	0.237	0.577	0.245	0.583	0.406	61.40%
Path 1: TSM to PC to MLA to AB	0.021	0.127	0.017	0.122	0.065	9.80%
Path 2: TSM to EC to MLA to AB	0.011	0.091	0.009	0.085	0.04	6.10%
Path 3: TSM to PC to AB	0.078	0.254	0.072	0.244	0.15	22.70%

TSM, Math Teacher Support; MLA, Math Learning Anxiety; PC, Problem-Focused Coping; EC, Emotion-Focused Coping; AB, Academic Buoyancy.

To further investigate the effect strengths of different mediation paths, this study used the Bootstrap method to conduct pairwise comparisons of the key indirect effects in the model. The labels for the three paths are shown in Table 4. The results revealed no significant difference between the mediating effects of Path 1 and Path 2 (Path 1-Path 2: [−0.047, 0.098]), nor between Path 1 and Path 3 (Path 1-Path 3: [−0.198, 0.003]). However, a significant difference was found between Path 2 and Path 3 (Path 3-Path 2: [0.030, 0.216]). Specifically, the pathway “Teacher Support → Problem-Focused Coping → Academic Buoyancy” was significantly stronger than the pathway “Teacher Support → Emotion-Focused Coping → Math Learning Anxiety → Academic Buoyancy”.

## 4 Discussion

### 4.1 Summary of research findings

The present study aimed to explore how math teacher support influences high school students’ academic buoyancy through the mediating roles of coping styles and anxiety. By constructing competitive models and chain mediation models, this study elucidated the specific pathways through which external support is structurally linked to psychological resilience. The core findings are summarized as follows:

First, this study clarified the differential impacts of distinct types of math anxiety on resilience. The findings supported the hypothesis that process-oriented math learning anxiety serves as the primary inhibiting factor for academic buoyancy, whereas outcome-oriented math evaluation anxiety no longer showed a significant effect after controlling for the former. This result indicates that a key underlying factor hindering high school students’ recovery from daily setbacks lies in cognitive difficulties and fear regarding the mathematical content itself, rather than mere concerns about external evaluations or test scores.

Second, math teacher support was identified as a critical protective resource for building student resilience. This protective effect occurs not only directly but also through its differential associations with students’ coping mechanisms. Specifically, teacher support enhances students’ ability to overcome difficulties via a direct mediation path that encourages the adoption of problem-focused coping; simultaneously, it indirectly protects buoyancy by being negatively related to negative emotion-focused coping, which in turn is associated with lower math learning anxiety.

Finally, a comparison of effect sizes for the mediation pathways further revealed that the effect of the independent mediation path “Teacher Support → Problem-focused Coping → Academic Buoyancy” was significantly stronger than that of the chain pathways involving emotional regulation and anxiety buffering. This suggests that in the process of enhancing students’ psychological resilience, prompting students to take proactive actions to solve mathematical problems is more critical and effective than merely helping them regulate emotions or alleviate anxiety.

### 4.2 The relationship between math learning anxiety, math evaluation anxiety, and academic buoyancy

The results showed that process-oriented math learning anxiety (MLA) as the primary inhibiting factor for academic buoyancy, whereas outcome-oriented math evaluation anxiety (MEA) did not show a significant independent effect after controlling for the former. This finding presents a notable challenge to prevailing assumptions within high-pressure, exam-oriented education systems like China’s. In such contexts, it is a common belief that exam or evaluation pressure is the primary culprit for undermining students’ psychological buoyancy. Our data, however, suggests a more nuanced reality where the fundamental threat to daily buoyancy appears to be the stagnation of the learning process rather than the periodic pressure of evaluation outcomes.

Specifically, math learning anxiety appears to be a process-oriented emotion that i permeates students’ daily listening, practicing, and thinking processes; According to the Encoding Deficit Hypothesis (EDH) (Eysenck and Calvo, 1992), when students experience high levels of anxiety during the information input stage, their limited working memory resources may be occupied by worrisome thoughts, potentially hindering the encoding process of new knowledge into long-term memory (Niaz and Logie, 1993; Chen and Geng, 2002; De Jong, 2010), impairing the individual’s learning ability, comprehension, and problem-solving skills in relevant contexts (McMullan et al., 2012). Furthermore, math learning anxiety is often associated with behavioral disengagement and avoidance during the daily acquisition of complex knowledge (Dowker et al., 2016). Since mathematics is a highly cumulative discipline, such avoidance at the encoding stage may fundamentally weaken the cognitive foundation required for buoyancy.

Notably, the non-significant independent effect of math evaluation anxiety appears somewhat counter-intuitive when contrasted with extensive Western literature, which frequently identifies test anxiety as a primary risk factor for academic resilience (Putwain et al., 2015, 2023). However, this can be explained through both cognitive and cultural lenses. Cognitively, while Attentional Control Theory (ACT) (Eysenck et al., 2007; Derakshan and Eysenck, 2009) suggests that evaluation anxiety consumes cognitive resources, its destructive effect is inherently situational (Eysenck et al., 2007). It acts primarily as an episodic disturbance to the output or externalization of existing knowledge during high-stakes exams, rather than disrupting the foundational, daily construction of knowledge itself. Therefore, the cognitive foundation for resilience remains relatively intact. Culturally, this episodic impact may be further attenuated by cultural habituation (Leung, 2006). Within the Chinese high school education system, characterized by high-frequency assessments, students may gradually develop a degree of psychological tolerance to evaluation pressure. Consequently, the episodic worry concerning test outcomes might no longer serve as the primary risk factor for their daily academic buoyancy (Martin et al., 2017).

This conclusion suggests that students’ “vulnerability” in mathematics stems more from the fear and avoidance behaviors triggered by the daily process of “not understanding” or “not being able to learn” the content, rather than solely from external evaluation pressure. This implies that future research exploring the mechanisms of emotional factors on resilience should more carefully consider the specific directionality of anxiety. Consequently, the focus of educational interventions might consider shifting from alleviating pre-exam nervousness to addressing cognitive barriers and emotional distress in the daily learning process. By lowering learning thresholds and providing cognitive scaffolding, educators can rebuild students’ sense of academic control at the source (Putwain et al., 2024), thereby protecting their academic buoyancy.

### 4.3 The relationship between teacher support and academic buoyancy

The results of this study confirm that math teacher support is significantly and positively associated with high school students’ academic buoyancy. This finding aligns with the conclusions of Abaszadeh et al. (2024) across different cultural contexts, indicating that external support from subject teachers is a vital resource for high school students in constructing internal psychological resilience during high school.

On one hand, instrumental support provided by math teachers (i.e., cognitive support and autonomy support) directly reduces the operational difficulty of objective tasks, acting as a “cognitive scaffold” for students coping with setbacks (Granziera et al., 2022). According to Conservation of Resources (COR) Theory (Hobfoll, 1989, 2001), individuals strive to acquire and maintain resources when facing stress. The discipline of mathematics involves a high degree of abstraction and logical rigor, making it a frequent source of academic frustration. When math teachers provide clear instructional guidance, timely feedback, and autonomy support, they are effectively transmitting cognitive resources for problem-solving directly to students (Alarcon et al., 2011). This injection of resources increases the likelihood that when students face daily predicaments of “not understanding” or “being unable to solve problems,” they do not rely solely on their own limited capabilities; instead, they can leverage the teacher’s professional strength as a buffer. This directly reduces the perceived threat of setbacks, thereby maintaining their level of buoyancy.

On the other hand, emotional support from math teachers, such as acceptance, care, and encouragement, can significantly enhance students’ sense of belonging and psychological safety (Romano et al., 2021). In mathematics, a subject associated with high anxiety, teacher emotional support conveys the signal that “errors are permissible, and difficulties can be shared”. This sense of psychological safety allows students to avoid activating defensive mechanisms to cover up mistakes when experiencing failure (e.g., poor test results, incorrect answers). Instead, they are able to maintain psychological flexibility, quickly recover their state, and re-engage in learning. Math teachers not only provide emotional comfort but also directly address the core problems inducing stress (e.g., obstacles in problem-solving). This “targeted” support model explains why math teacher support serves as a significant predictor of student buoyancy.

### 4.4 The relationships among coping styles, math anxiety, teacher support behavior, and academic buoyancy

Based on the Protective Factor Model (Garmezy et al., 1984), this study deeply explored the internal mechanisms through which teacher support influences academic buoyancy. The findings revealed that math teacher support is linked to students’ buoyancy levels not only through the direct mediating role of problem-focused coping but also through a chain mediating effect involving emotion-focused coping and math learning anxiety. This finding resonates with the Protective Factor Model (Garmezy et al., 1984), reflecting that the protective function does not simply eliminate risk but rather operates through a layered process of transformation (Garmezy et al., 1984). Teacher support first alters students’ sense of control and value appraisal regarding the situation, prompting them to adopt more adaptive coping behaviors (Lazarus and Folkman, 1984). This, in turn, is linked to lower levels of interference from math learning anxiety, a risk factor on academic buoyancy. This demonstrates that teacher support, as an external protective factor, buffers and transforms the impact of internal risk factors by influencing students’ behavioral choices and emotional regulation, ultimately contributing to the construction of internal psychological resilience.

Notably, a comparison of the effect strengths across pathways revealed that the effect of the “Teacher Support → Problem-focused Coping → Academic Buoyancy” pathway was significantly stronger than that of the “Teacher Support → Emotion-focused Coping → Math Learning Anxiety → Academic Buoyancy” pathway. The former represents a “value-added” adaptive process, while the latter is a “buffering” chain pathway, implying that the efficacy of proactive action is significantly superior to mere emotional regulation. In the pathway mediated by problem-focused coping, the support provided by teachers satisfies students’ basic psychological needs and ignites their intrinsic motivation (Reeve, 2012). This encourages students to view stressors as solvable problems, thereby actively adopting problem-focused coping strategies (He, 2012; Raine et al., 2025). Problem-focused coping is an active adaptation style aimed at changing the environment (Carver et al., 1989), manifested as students actively analyzing errors or seeking help from peers and teachers. Each successful problem-solving experience resulting from personal effort accumulates daily academic self-efficacy and a sense of control (Lei et al., 2022), and this positive construction facilitates the “value addition” of buoyancy. Conversely, in the mediation pathway involving emotion-focused coping, while teacher care and encouragement can reduce avoidance or denial behaviors stemming from helplessness and buffer the impact of negative emotions, this approach—which suppresses anxiety by reducing negative behaviors—does not directly provide students with problem-solving tools or enhance their resolution capabilities; it merely protects buoyancy from depletion. This suggests that in the construction of students’ academic buoyancy, the focus of teacher support should be placed on inspiring and cultivating students’ willingness and ability to actively solve problems. Furthermore, the study found that when math learning anxiety is present, the direct mediating effect of emotion-focused coping was not significant, which diverges from our hypothesis and some previous studies (Tolan et al., 2002; Meneghel et al., 2019). To interpret this finding accurately, it is crucial to move beyond a strict binary view of coping strategies. Emotion-focused coping is not a uniformly negative construct; it encompasses both maladaptive strategies (e.g., avoidance, catastrophizing, rumination) and adaptive ones (e.g., emotional approach coping, cognitive reappraisal) (Folkman and Moskowitz, 2004). The non-significant or negative indirect effect found in our study likely reflects the specific demands of the discipline: in a subject like mathematics, which presents absolute cognitive barriers, mere low-level emotional venting cannot resolve substantive knowledge gaps. Instead, by consuming limited cognitive resources, these maladaptive emotional responses can catalyze math learning anxiety, thereby indirectly eroding students’ academic buoyancy (Michl et al., 2013).

However, this outcome does not imply that emotional regulation lacks value. High-level adaptive emotional strategies, such as cognitive reappraisal, reframing academic frustration as an inevitable “growing pain” in intellectual development rather than an ego threat, remain vital for maintaining psychological flexibility (Meneghel et al., 2019; Pekrun, 2024). Ultimately, while resolving cognitive barriers via problem-focused coping is paramount in mathematics, guiding students to employ high-order emotional regulation can synergize with proactive problem-solving to effectively enhance their academic buoyancy.

### 4.5 Theoretical significance and possible directions

The theoretical value of this study is twofold. First, it explicitly distinguishes between math learning anxiety and math evaluation anxiety through empirical data, revealing that the former is the core risk factor influencing high school students’ academic buoyancy. This contributes to a deeper theoretical understanding within the field of academic emotions. Second, based on the Protective Factor Model (Garmezy et al., 1984), this study constructs and validates a chain mediation model. This model elucidates the internal psychological pathways through which teacher support, as an external protective factor, correlates with students’ choice of coping styles, subsequently influencing their levels of math learning anxiety, and ultimately acting upon their academic buoyancy. This provides procedural evidence for understanding how protective factors function in the presence of risk factors.

The findings of this study provide empirical evidence for integrating social–emotional learning (SEL) principles into daily mathematics instruction. We recommend that school psychological services should transcend the traditional model of individual emotional counseling and transition toward an “instructional consultation” model characterized by deep collaboration with subject teachers. This approach aims to reduce anxiety and reshape coping mechanisms at the source by optimizing instructional design. Specifically, the findings hold significant implications for high school educational practice:

First, educators might consider shifting the focus of support from alleviating “grade anxiety” to overcoming “content anxiety.” Given that Math Learning Anxiety stems primarily from the preemption of limited working memory resources by the cognitive processes required for specific content, school psychologists should guide math teachers to integrate the principles of Cognitive Load Theory into daily instruction. For students with high levels of anxiety, mere pre-exam relaxation training often treats the symptoms rather than the root cause. Teachers should be encouraged to adopt strategies such as “worked examples” and “fading scaffolding” when explaining highly abstract concepts. For instance, teachers can provide demonstrations of complete problem-solving steps and gradually withdraw scaffolding as students’ proficiency increases, thereby reducing the “extraneous cognitive load” of the task. This practice of lowering cognitive thresholds through instructional design allows students to gain an immediate sense of competence within micro-learning units. It effectively blocks the vicious cycle of “incomprehension—anxiety—avoidance” at its source, which may be a more effective strategy than retroactive emotional soothing.

Second, future interventions may benefit from relieving negative emotions to cultivating proactive adaptive behaviors. Our research indicates that the most effective pathway for teacher support lies in inspiring students to actively solve problems, rather than merely buffering negative emotions. We recommend that school psychologists assist teachers in embedding “Attribution Retraining” into their classroom feedback. When students encounter daily setbacks in problem-solving, teachers should guide them to avoid attributing failure to stable deficits in ability (e.g., “I am naturally bad at math”). Instead, failure should be reframed as specific, resolvable technical errors or strategic deficiencies (e.g., “I need a new method for this derivation step”). Furthermore, to address the overwhelming pressure imposed by mathematical tasks, teachers should instruct students in the use of “task decomposition” strategies. Students should be guided to break down vague and monolithic academic threats (e.g., “I cannot master this chapter”) into a series of actionable micro-challenges (e.g., “I will first understand this single definition”). By transforming abstract anxiety into concrete action plans, students can gradually rebuild their sense of control over the learning process, thereby facilitating a spontaneous shift from passive emotion-focused coping to active problem-focused coping.

### 4.6 Limitations

Despite the theoretical and empirical contributions of this study, three limitations should be acknowledged to contextualize the findings and guide future research.

First, the cross-sectional design limits causal inference. Although the structural equation modeling provided statistical support for the hypothesized directional paths, the use of cross-sectional data precludes the definitive determination of temporal precedence between variables. In authentic educational settings, complex reciprocal effects (bidirectional causality) are highly likely to exist. For instance, while our model posits that teacher support facilitates problem-focused coping and buffers anxiety, an inverse dynamic may also be at play: students with inherently higher academic buoyancy might proactively employ problem-focused coping, which in turn elicits more instrumental and emotional support from their teachers. Conversely, students paralyzed by severe math learning anxiety might display withdrawal behaviors that inadvertently discourage teacher-student interactions, creating a negative feedback loop of “low support–high anxiety–low buoyancy”. Future research should employ longitudinal cross-lagged panel models (CLPM) or daily diary designs to meticulously disentangle these dynamic, bidirectional relationships over time.

Second, the cultural and regional context of the sample limits the external validity of the findings. The participants were recruited primarily from high schools in an economically developed region of Eastern China, an environment characterized by high academic competition and a strong societal emphasis on the national college entrance examination. In this specific educational ecosystem, the profound expectations for academic excellence and the pressure associated with high-stakes assessments may uniquely amplify the dynamics between content-oriented anxiety and academic buoyancy. Consequently, caution should be exercised when generalizing these findings to students from less competitive educational systems. Future research should expand the sampling scope to include cross-regional or cross-cultural comparisons to test the universality of this theoretical model across different educational ecologies.

Third, the subject specificity may limit the cross-disciplinary applicability of the theory. This study specifically selected mathematics, a subject characterized by high cognitive load and prone to inducing process-oriented anxiety. However, the mechanisms triggering anxiety and building resilience may differ across disciplines. Therefore, whether the current findings apply to other subjects requires further verification. Future research should compare the mechanisms of teacher support and resilience across different subject contexts to enrich the domain-specificity theory of academic emotions and adaptation.

Fourth, all variables in this study were assessed utilizing student self-report questionnaires. Although rigorous statistical tests (e.g., the ULMC approach) indicated that common method bias did not substantially distort the results, subjective self-reports are inherently susceptible to social desirability and recall biases. Future studies could incorporate multi-informant approaches, such as teacher evaluations, behavioral observation data, or daily diary methods, to obtain a more objective and comprehensive assessment of students’ academic resilience and coping mechanisms.

### 4.7 Conclusion

(1) Math teacher support not only shows a direct positive association with academic buoyancy but also positively predicts promoting problem-focused coping (and inhibiting emotion-focused coping), thereby being associated with lower math learning anxiety.
(2) Math learning anxiety significantly and negatively predicts academic buoyancy, whereas the impact of math evaluation anxiety is not significant.
(3) The indirect effect of the path “Teacher Support → Problem-Focused Coping → Academic Buoyancy” is significantly stronger than the indirect effect of the path “Teacher Support → Emotion-Focused Coping → Math Learning Anxiety → Academic Buoyancy”.

## Data Availability

The raw data supporting the conclusions of this article will be made available by the authors, without undue reservation.
